# Role of Nitric Oxide and Protein S-Nitrosylation in Ischemia-Reperfusion Injury

**DOI:** 10.3390/antiox11010057

**Published:** 2021-12-27

**Authors:** Hyang-Mi Lee, Ji Woong Choi, Min Sik Choi

**Affiliations:** 1 College of Pharmacy, Dongduk Women’s University, Seoul 02748, Korea; hmlee@dongduk.ac.kr; 2 College of Pharmacy, Gachon University, Incheon 21936, Korea; 3 Laboratory of Pharmacology, College of Pharmacy, Dongduk Women’s University, Seoul 02748, Korea

**Keywords:** ischemia-reperfusion injury, nitric oxide, protein S-nitrosylation

## Abstract

Ischemia-reperfusion injury (IRI) is a process in which damage is induced in hypoxic tissue when oxygen supply is resumed after ischemia. During IRI, restoration of reduced nitric oxide (NO) levels may alleviate reperfusion injury in ischemic organs. The protective mechanism of NO is due to anti-inflammatory effects, antioxidant effects, and the regulation of cell signaling pathways. On the other hand, it is generally known that S-nitrosylation (SNO) mediates the detrimental or protective effect of NO depending on the action of the nitrosylated target protein, and this is also applied in the IRI process. In this review, the effect of each change of NO and SNO during the IRI process was investigated.

## 1. Introduction

Nitric oxide (NO) participates in and regulates several pathological or physiological processes, including cell proliferation, differentiation, and inflammatory responses [1]. Protein S-nitrosylation (SNO), also known as NO-mediated reversible protein modification, alters the activity, function, and subcellular localization of a target protein [2]. A series of recent studies have also uncovered novel mechanisms of NO regulation by discovering protein–protein transnitrosylation reactions (movement of NO groups from one protein to another) [3,4]. On the other hand, the level of S-nitrosoglutathione (GSNO) and other S-nitrosothiols is regulated by GSNO reductase (GSNOR), a major regulator of NO/SNO signaling [5].

Ischemia limits the blood supply to certain tissues, resulting in decreased supply of glucose and oxygen to the tissues of the brain, heart, liver, lungs, or kidneys [6]. Although ischemia itself is reversible when the blood is resupplied to the affected tissue, it induces a secondary damage known as ischemia-reperfusion injury (IRI). IRI is marked by increased production of reactive oxygen species (ROS) and proinflammatory factors that cause severe tissue damage [7,8]. In this paper, recent studies on the relationship of NO, SNO, and GSNOR in the IRI process are reviewed, and the potential of the related signal transduction system as a therapeutic target is investigated (Table 1).

## 2. Ischemia-Reperfusion Injury and Nitric Oxide

### 2.1. Ischemia-Reperfusion Injury

Ischemia-reperfusion injury (IRI) is induced by multifaceted cellular processes in hypoxic tissues after return of oxygen delivery, which leads to a dysfunction of organs such as the brain and heart, resulting in ischemic stroke and myocardial infarction, respectively. In ischemia stage, the blockage of blood flow causes a loss of homoeostatic function of cells due to hypoxia and dysfunction of mitochondria. In contrast to an assumption that prompt perfusion is beneficial to preserve cellular function, a vast array of studies suggest that reperfusion potentially aggravates injury in ischemic cells. During reperfusion with recovered blood flow, oxygen provided to hypoxic cells promotes the generation of reactive oxygen species (ROS) that induce oxidative stress in critical enzyme systems involving NADPH oxidase and nitric oxide synthase (NOS) systems. Oxidative stress subsequently induces cell damage and then cell death, resulting in IRI [7]. In particular, the activity of NOS is decreased due to oxidization in IRI, leading to a reduction of NO [33]. Thus, a lowered level of NO accompanied with accumulation of ROS may contribute to IRI-induced cell death.

### 2.2. Nitric Oxide (NO) Pathophysiology in Ischemia-Reperfusion Injury

NO is a molecule with a short half-life in the form of gas that is permeable of cell membranes. In biological tissues, NO is produced from L-arginine by a group of NO synthases (NOS) comprised of three isoforms: endothelial NO synthases (eNOS or NOS3), neuronal NO synthases (nNOS or NOS1), and inducible NO synthases (iNOS or NOS2). eNOS and nNOS are constitutively expressed, generating a low level of NO. On the other hand, iNOS can produce a large amount of NO once induced mainly in inflammatory cells, for example, macrophages and astrocytes [34]. iNOS has been reported to catalyze NO production by induction of lipopolysaccharides (LPS), interleukin-1 (IL-1), and tumor necrosis factor (TNF) in inflammatory conditions [35].

One way by which NO exerts its function is via direct interaction with various proteins. This can result in disulfide bonds between cysteine and glutathione [36], or protein nitrosylation [37]. In addition, NO is known to be able to activate cell signaling by promoting cyclic guanosine monophosphate (cGMP) generation.

NO plays a diverse role in tissues including muscular or neuronal cells. It was described that NO generated by endothelial NOS moves to neighboring smooth muscle cells and activates cGMP-induced phosphorylation of kinases, resulting in relaxation of vascular smooth muscle cells [38]. NO also affects various functions of neuronal cells, such as their proliferation and differentiation, as well as neurogenesis in the adult brain [39,40,41,42].

Along with its role in normal physiological status as summarized above, NO is also a critical mediator in cellular disorders caused by dysregulation of oxygen supply and inflammation. It was demonstrated that a reduced production of NO is due to a compromised activity of eNOS during IRI [43]. In stroke, ischemia of brain tissue induces changes in the concentration of NO. eNOS and nNOS exhibit a profound reduction in their activity in an ischemic setting [44]. In accordance with this, previous reports have shown that a largely reduced NO level lasted in the ischemic brain upon middle cerebral artery occlusion [45,46], followed by a transient increase of NO concentration after reperfusion [47]. Given this pathophysiology of NO in IRI, extensive studies have been conducted to elucidate the effect of NO on protection of tissue injury. Furthermore, varying approaches, such as a delivery of exogenous NO, have been suggested to limit reperfusion-mediated ischemic tissue injury.

Importantly, the opposite effect of NO on IRI is also observed. For example, it has been reported that reperfusion increased nNOS and iNOS expression and NO production after focal cerebral ischemia, which may also have detrimental implications through direct activation of matrix metalloprotease-9 (MMP-9) [48]. This suggests limitations and possible side effects of NO-based therapeutics for IRI, which will be discussed next.

### 2.3. Role of Nitric Oxide in Protection of Ischemia Reperfusion Injury

**Figure 1:**
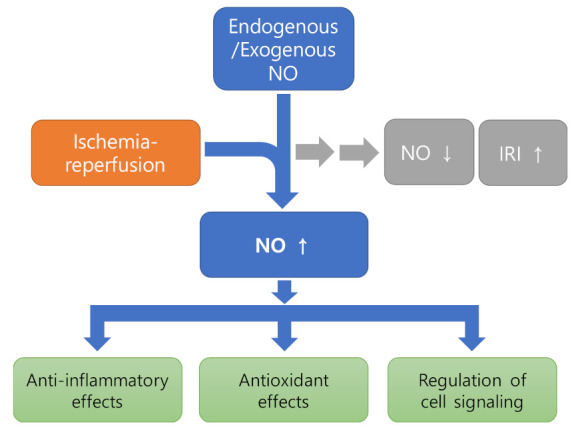
Diagram showing the protective effects of NO against IRI by modulating various aspects of cellular function. It can be seen that endogenous or exogenous NO restores NO reduction and inhibits further progression of IRI.

Ample studies suggest that restoration of NO during ischemia is able to limit reperfusion damage of tissues such as the brain [49], heart [50], and liver [51], establishing a current notion of the protective role of NO in IRI. The protective effect of NO can be exerted thorough regulating various aspects of cellular function (Figure 1).

#### 2.3.1. Anti-Inflammatory Effects

Administration of NO appears to regulate diverse inflammatory responses in ischemia-related injury. First, previous reports indicated that NO has downregulated inflammatory cytokines or chemokines. Exogenous NO reduced cytokine expression, which led to a diminished injury of the liver [52]. Among pro-inflammatory cytokines, the contribution of TNFα or IL-1 to IRI and the protective effect of NO have been well known. TNFα or IL-1 appeared to act as a stimulator that enhances the infiltration of neutrophils in liver IRI [53]. TNFα was also capable of enhancing synthesis of chemokines in ischemic tissues via activation of NF-κB [54]. NO donors were demonstrated to be effective in reduction of pro-inflammatory cytokines including TNFα in IRI of the kidney or liver [9,10]. A previous study using iNOS^−/−^ mice also revealed the effect of NO on protecting hepatocytes from TNFα-induced apoptosis [55]. Additionally, IL-1 was inhibited by NO in IRI induced by liver graft [11].

Regarding the role of chemokines in IRI, ischemia reperfusion was shown to activate the expression of macrophage inflammatory protein-1 (MIP-1) and -2 (MIP-2), which enhanced neutrophil infiltration [12]. The protective role of NO in IRI is attributed to its capability to suppress MIP expression by activating several signaling pathways including MAPK (ERK1/2) and NF-κB [56,57].

Moreover, NO could prevent the infiltration of inflammatory cells, such as neutrophils, into the ischemic tissue, thereby attenuating injury of the lung, heart, liver, or kidney [58,59,60,61]. NO exerts its inhibitory effect on immune cell recruitment by influencing adhesion molecules. A varied array of inflammatory mediators including cytokines and chemokines can stimulate the induction of cell adhesion molecules (CAM) in ischemia and reperfusion conditions [62]. For instance, an increased level of selectins (P- or L-selectin) was shown to be involved in the attachment of neutrophils to the endothelium thorough mediating rolling and adhesion of inflammatory cells in the liver IRI settings [63,64]. In addition to the previous report suggesting the inhibitory effect of NO donors on P-selectin expression [13], it has been demonstrated that NO donors also suppressed NF-κB activation and the induction of other CAMs, such as E-selectin, vascular cell adhesion molecule-1 (VCAM-1), and intracellular adhesion molecule-1 (ICAM-1) in the endothelial cells [65,66].

#### 2.3.2. Effect as Antioxidant

NO has been shown to act as a radical scavenger by interacting with superoxide, which generates peroxynitrite [14]. NO was also suggested to have a radical scavenger effect in the ischemic heart [15]. In addition, NO is known to inhibit mitochondrial respiration, which leads to a reduction of reactive oxygen species (ROS) generation [16,17]. In the ischemic condition with lack of oxygen supply, NO likely outcompetes oxygen for binding to cytochrome-c oxidase in the electron transport chain, and thereby produces less ROS when reperfusion occurs [18].

#### 2.3.3. Regulation of Cell Signaling

NO has been shown to modulate various signaling pathways, which promotes a protection of tissues from IRI. In particular, pro-inflammatory mediators such as TNFα have exhibited an activation of mitogen-activated protein kinases (MAPKs), involving extracellular signal-regulated kinases (ERK), c-Jun N-terminal kinases (JNK) and p38 kinases, during ischemia and reperfusion [67], implying a potential protection from IRI by inhibition of these signals. On the other hands, these MAPKs have also been shown to be activated by NO and to participate in NO-mediated signaling [68]. An application of NO has exhibited beneficial effects through activation of the p38 MAPK pathway, preventing injury of hepatocytes [19]. One mechanism by which NO manipulates downstream MAPK pathways is the nitrosylation of the thiol residues, which will be further discussed in this review.

Other than MAPKs, NO has been well known to inhibit transcription factors such as NF-κB and AP-1, which contribute to IRI. Early reports indicated that NO mediated suppression of NF-κB-regulated endothelial CAM expression [69]. It has been demonstrated that NO employs varying mechanisms for the inhibition of NF-kB function. NO decreased the binding of NF-κB to DNA through either inducing the stabilization and gene expression of IκBα or a direct modification of NF-κB, such as nitrosylation of cysteine residue [20]. Moreover, NO attenuated the activity of NF-κB by removal of a superoxide radical that has been shown to activate NF-κB [21]. AP-1 also contributes to IRI via an involvement of cytokine-induced or apoptotic signaling [22]. Attenuation of AP-1 and subsequent apoptosis of neuronal cells is attributed to NO [23].

### 2.4. Therapeutic Approaches

#### 2.4.1. Direct Administration of NO

A NO gas product (INOmax) for inhalation was initially approved by the FDA in 1999 for the treatment of neonates with a hypoxic respiratory defect from pulmonary hypertension to improve oxygenation. Since then, mounting evidences has suggested NO inhalation as a beneficial approach in multiple organ dysfunctions associated with IRI [70]. Inhalation of NO before and during reperfusion has been shown to reduce cardiac IRI [71,72]. In a cerebral ischemia model, supply of inhaled NO exhibited a significant protective effect on brain tissue damage, with reduced lesion size but improved behavioral function [50]. A protective effect of inhaled NO was demonstrated in experimental subarachnoid hemorrhage, which led to reduced brain damage accompanied with increased survival rate [73]. As for clinical study of NO inhalation, brief administration of inhalative NO resulted in a hemodynamic improvement in right ventricular myocardial infarction and cardiogenic shock [74]. In a recent trial, efficacy of NO inhalation was not observed in patients with ST-elevation myocardial infarction, in which infarct size remained similar after NO treatment [75].

#### 2.4.2. NO Donors

To provide NO in its deficiency after ischemia and reperfusion, a variety of NO donors have been applied in both preclinical and clinical studies. Examples of exogenous NO donors involve glyceryl trinitrate (GTN), sodium nitroprusside (SNP), and sodium nitrite. Following the early success of NO donors on amelioration of kidney and liver injuries in IRI settings in the rat [76,77], analysis of multiple studies on experimental stroke also indicated a neuroprotective role of NO donors [78]. With encouraging preclinical data, clinical trials for ischemic stroke have been conducted by using a transdermal GTN patch, which deliver GTN without affecting cerebral blood flow. In an Efficacy of NO in Stroke (ENOS) trial, transdermal GTN improved the functional index of blood and pulse pressure in acute stroke [79,80]. On the other hand, in the recent Rapid Intervention with GTN in Hypertensive stroke Trial-2 (RIGHT-2), transdermal administration of GTN exhibited no efficacy in highly-acute stroke patients [81]. Regarding myocardial IRI, animal studies have suggested an overall positive effect of NO donors while clinical trials generated variable results [82]. A measurement of plasma NO levels has shown severely reduced NO in patients with acute myocardial infarction. Based on this finding, a trial was conducted using a SNP pad to increase systemic NO levels in cancer patients, who generally have higher risk of death caused by acute myocardial infarction than the normal population. Of interest, application of SNP pads for several years increased the plasma NO concentration to a normal level, resulting in a prevention of death from acute myocardial infarction [83]. Nonetheless, administration of sodium nitrite has shown no change in infarction size. In a trial with acute ST-elevation myocardial infarction patients, immediate I.V. infusion of sodium nitrite before reperfusion did not reduce infarction size [84]. Similarly, sodium nitrite administrated via intracoronary injection did not change infarction size despite a largely improved myocardial index and fewer adverse events [85].

#### 2.4.3. Advances in NO Delivery System

The application of NO gas as a therapy has its intrinsic limit, including short half-life and uncontrolled targeting. Thus, solidified NO in the context of NO donors developed in various forms such as organic nitrates, S-nitrosothiols, and NO-releasing biomaterials. A recent study showed that a high-density lipoprotein (HDL)-like particle, comprised of synthetic S-nitrosylated phospholipid and proteins of HDL, has functions of both NO and HDL, which provide efficacy and specific targeting, respectively. This synthetic bioparticle demonstrated a reduced IRI in a mouse kidney transplant mode in vivo [86]. Moreover, recent advances have been made in nanotherapy, which enable a targeted delivery of NO to ischemia reperfusion-injured tissues. It was also demonstrated that S-nitrosothiol coated paramagnetic nanoparticles (SNO-PMNPs) improved reflow and functional capillary density while preventing cell death in the presence of a magnet in animal IRI setting, indicating a potential usage of magnetic-field-induced delivery of NO to treat localized ischemic diseases [87].

## 3. Ischemia Reperfusion Injury and Protein S-Nitrosylation

### 3.1. NO and Protein S-Nitrosylation

It is believed that the biological action of NO in the cell is mainly mediated through guanylate cyclase activation and subsequent production of cyclic guanosine-3′, 5′-monophosphate (cGMP). In addition to this, S-nitrosylation, a covalent reaction between the reactive cysteine thiol of the target protein and the NO group, has appeared as another important mechanism for the biological function of NO [88]. It is known that the formation of S-nitrosoproteins (SNO-proteins) results in allosteric or direct modification of the active site cysteine, which in turn modulates protein function [88,89]. Currently, more than 3000 proteins have been identified as potential target proteins that can be S-nitrosylated to cause structural and functional changes [90]. The functional changes of most SNO-proteins are useful for further pathophysiological studies, supporting the conclusion that NO exerts significant intracellular activity through the S-nitrosylation process. NO derived from NOS induces the formation of SNO-proteins by rapidly and efficiently S-nitrosylating adjacent proteins. Working with proteins located near NOS, NO also reacts with cysteine itself and with glutathione (GSH), which in turn can form low-molecular-weight SNO-molecules. These SNO-molecules correspond to S-nitrosocysteine (CSNO) and S-nitrosoglutathione (GSNO), respectively. Depending on their intrinsic redox potential, they can act as NO donors to other proteins in the physiological environment [88]. In fact, until recently, transnitrosylation, defined as the transfer of NO from one thiol to another, was believed to occur only between the low-molecular-weight SNO-protein and the target protein thiol. However, several groups in this field have shown that transnitrosylation between functional proteins within cells may be another enzymatic activation process involving S-nitrosylation [91,92,93,94,95,96,97].

### 3.2. Role of SNO-Proteins in Ischemia Reperfusion Injury

**Figure 2:**
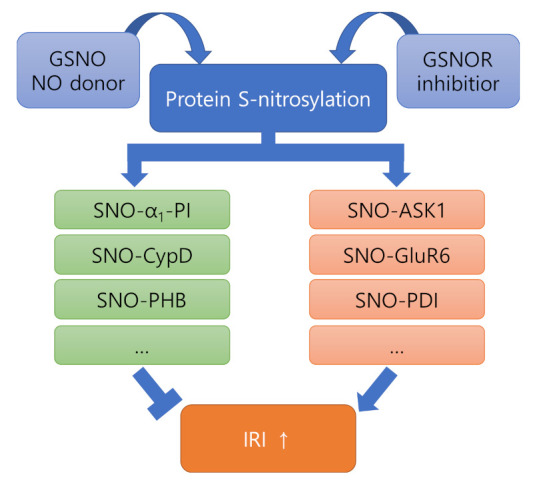
The role of S-nitrosylation in IRI. S-nitrosylation causes a functional change of the nitrosylated target protein, which tends to inhibit or promote severe progression of IRI. This suggests that inhibition of SNO-proteins in the orange box should be considered for more efficient use of NO donors or GSNOR inhibitors for the treatment of IRI.

In general, it is known that S-nitrosylation mediates deleterious or protective effects of NO depending on the action of the nitrosylated target protein [2]. Therefore, we will summarize the studies conducted in relation to IRI from the perspective of NO/SNO signaling and examine whether the functional ambivalence of NO/SNO signaling is also reflected in IRI (Figure 2).

#### 3.2.1. Proteins Showing Negative Effects after Nitrosylation (ASK1, GluR6, PDI)

Apoptotic signal-regulated kinase 1 (ASK1) is one of the mediators of cell death induced by stimuli including reactive oxygen species, excessive calcium influx, and ischemia [98]. Phosphorylation of ASK1 at Thr845 is associated with ASK1 activation and consequent ASK1-dependent apoptosis [28]. By S-nitrosylation, the level of ASK1 phosphorylation at Thr845 causing ASK1 activation was increased [29]. In this paper, it was also confirmed that S-nitrosylation not only induced an increase in ASK1 dimerization, but also activated JNK signaling and the following nuclear apoptotic pathways during the initial stage of IRI, which is involved in nerve damage.

Another protein that is nitrosylated and mediates the progression of IRI is glutamate receptor 6 (GluR6) [30,31]. The synthesis of NO by nNOS in the brain is mainly induced by intracellular calcium influx through N-methyl-D-aspartate (NMDA)-type glutamate receptors forming a postsynaptic protein complex with nNOS compiled by postsynaptic density protein 95 (PSD95) [99]. Consistent with this, it has been reported that the generation of endogenous NO could be mediated by the NMDAR-PSD95-nNOS signaling pathway during the early stage of IRI [31]. GluR6 S-nitrosylation was induced by endogenous NO, and NOS inhibitors markedly suppressed SNO-GluR6 generation. In addition, when the S-nitrosylation level of GluR6 was reduced, the binding of PSD95 to GluR6 and mixed lineage kinase 3 (MLK3) and the JNK signaling pathway were downregulated. Accordingly, a protective effect was induced against apoptosis of hippocampal CA1 in cerebral ischemia.

Protein disulfide isomerase (PDI), an ER chaperone protein, is important for normal and proper protein folding. PDI is primarily responsible for forming the structural stability of proteins through the formation of disulfide bonds [100]. S-nitrosylation of PDI has been reported, which may result in the formation of aggregates of ubiquitinated proteins [32]. In this study, it was demonstrated that cultured astrocytes can induce iNOS upregulation and excessive NO production in response to IRI stress. Eventually, SNO-PDI becomes functionally impaired, suggesting a deep link between free radical production and aberrant protein aggregation in cerebral IRI.

#### 3.2.2. Proteins Showing Positive Effects after Nitrosylation (α1-PI, CypD, PHB)

It has been reported that the α1-protease inhibitor (α1-PI), a protease inhibitor in human plasma, is nitrosylated to form SNO-α1-PI in IRI of rat liver tissue, thereby showing cytoprotection [24]. At this time, all SH groups of α1-PI were nitrosylated. By SNO-α1-PI treatment, plasma liver enzyme elevation, hepatic neutrophil accumulation and hepatocyte apoptosis were greatly reduced, and hepatic blood flow was improved. In this study, unfortunately, low-molecular-weight nitrosothiols such as GSNO did not show an obvious protective effect, but NOS inhibitors exacerbated liver IRI as expected.

Although the cardioprotective effect of nitroglycerin administered before ischemic injury is well known, it remains unclear whether and how acute low-dose administration of nitroglycerin after ischemic injury limits infarct size. As an answer to this, in a study on the post-ischemic protective effect of nitroglycerin, it was confirmed that the action of nitroglycerin is due to the inhibition of mitochondrial permeability transition pore (mPTP) opening by S-nitrosylation of cyclophilin D (CypD) induced by eNOS [25]. Interestingly and conversely, ischemic heart biopsies showed decreased eNOS enzyme activity and reduced SNO-CypD formation [25].

Prohibitin (PHB) is a major mitochondrial protein with neuroprotective effects when upregulated in mice, but the mechanism by which PHB regulates protective functions during IRI is not well understood [26]. As a mechanism of the protective effect of prohibitin, it was confirmed that PHB and NO directly interact to cause protein S-nitrosylation at Cys69 of PHB, thereby having a protective effect [27]. In this study, it was found that for PHB to have a protective effect, an appropriate increase in protein levels is required and the interaction with NO is also essential. In addition, it was confirmed that PHB nitrosylation can be induced by NOS-dependent neuronal synaptic activity.

### 3.3. Role of GSNOR in Ischemia Reperfusion Injury

As a major NO donor in physiological systems, GSNO has an intrinsic metabolic process, in which GSNO becomes a selective substrate for GSNOR, whereby it is completely reduced to glutathione disulfide (GSSG) and ammonia. Since GSNO is in equilibrium with other SNO-proteins within the cell, GSNO metabolism by GSNOR indirectly regulates the overall intracellular SNO-proteins and the related signaling mechanisms [101]. On the other hand, some other reducing enzymes have been reported to metabolize SNO-proteins in vitro [102,103], but they have not been confirmed to regulate endogenous SNO levels.

The function and mechanism of action of GSNOR in IRI have been largely elucidated in ischemic heart disease-related studies. GSNOR plays its part in terms of systolic force and vascular tone for normal heart function [104]. GSNOR-deficient mice showed problems with calcium influx, resulting in decreased vascular resistance and β-adrenergic response [101], and as a result, GSNOR-deficient mice are protected from damage induced by myocardial infarction [105,106].

Very recently, it was reported that pharmacological inhibition of GSNOR protects the heart from IRI [107]. The protective effect on the heart was associated with increased SNO-proteins, and in particular, increased S-nitrosylation of mitochondrial complexes III and V was observed. Fortunately, in this study, a GSNOR inhibitor, 5-chloro-3-(2-[4-ethoxyphenyl) (ethyl) amino]-2-oxoethyl)-1H-indole-2-carboxylic acid (C2), did not show any adverse effects on cardiac function, including arrhythmias.

## 4. Effect of NO on the Comorbidities of Ischemic Stroke

Unlike childhood stroke, ischemic stroke in adults is primarily a disorder of vascular comorbidities and/or changes in blood coagulation status. As modernization progresses, lifestyle changes including dietary habits are contributing to the increase in stroke incidence [108]. Therefore, in the case of stroke, the presence of comorbidities is particularly important. In this section, we will examine the effect of NO on hypertension and atherosclerosis, which are major comorbidities of stroke.

### 4.1. Hypertension

Because high blood pressure is a major cause of cerebral small vessel disease (CSVD), the effect of hypertension on cerebral blood circulation is profound in stroke. Structural remodeling inside large and small arteries reduces lumen diameter and vasodilation capacity, and if this condition persists, it can lead to hypoperfusion and hemodynamic vascular damage [109]. In hypertensive conditions, repeated mechanical stress on the vessel wall and inadequate degradation of vascular elastin fibers stiffen the arteries and subsequently transfer the pulsatile load to the brain parenchyma [110,111].

Other adverse pathological processes associated with hypertension include endothelial dysfunction and decreased production of NO, which results in increased cerebrovascular resistance (CVR) and reduced autoregulatory capacity [112]. Additionally, hypertension also increases the shear stress applied to the vascular endothelial cells. Under normotensive conditions, this increased shear stress induces an adaptive vasodilation response through increased production of NO, which has the ability to renormalize shear stress [113,114]. Conversely, in hypertensive conditions that suppress NO production mechanisms, the lack of an adaptive response to elevated shear stress results in endothelial damage as well as upregulation of atherogenesis-related genes [113,114]. Altogether, this means that the increase in shear stress on the cerebral vascular endothelium induced in the hypertensive state causes atheroma formation and subsequent atherosclerosis, another comorbidity of stroke. It indicates that the inhibition of NO production by hypertension is an important basic risk factor for cerebrovascular occlusion.

### 4.2. Atherosclerosis

It has been reported that atherosclerosis and hypercholesterolemia inhibit endothelium-dependent NO-mediated regulation of vascular tone [115,116]. An even and appropriate distribution of NO is maintained in blood vessels under normal physiological conditions, which is determined and controlled by the occurrence of laminar or turbulent blood flow or changes in blood flow velocity. NO interferes with the interaction of the blood elements circulating within the vessels, most of the components involved in atherosclerosis, with the vessel walls. In other words, the adhesion and aggregation of platelets and monocytes to the vessel wall is inhibited by NO.

It is well known that endothelial dysfunction occurs as an early event in atherosclerosis and coronary heart disease (CHD) [117,118]. Dysfunction of the NOS signaling pathway may be one of the early events of atherosclerosis, suggesting that NO synthesis and/or decreased activity of NO may contribute to the initiation and progression of atherosclerosis [117,118,119]. NOS signaling derangement can be caused by several mechanisms, including (1) lipoprotein-induced alteration of signal transduction, (2) increased superoxide anion production followed by degradation of NO, (3) decreased affinity of NOS for L-arginine, and/or (4) increased levels of circulating antagonists against NOS [117,118,119].

From this point of view, strategies to enhance NO synthesis and/or NO activity may be useful for the treatment of atherosclerosis as well as hypertension as discussed above, and are expected to enable a new approach to the treatment of ischemic stroke.

## 5. Concluding Remarks

In general, NO is reduced during the IRI process, and a protective effect appears when the NO level is restored. However, as reviewed above, each SNO-protein can mediate protective or impairing processes during IRI. Therefore, in considering the therapeutic use of the abovementioned NO-donating reagents for IRI, the development of safer and more effective therapies will be possible only when the individual regulation of each SNO-protein is also considered.

## Figures and Tables

**Table 1:** Role of nitric oxide and protein S-nitrosylation and their targets in ischemia-reperfusion injury.

NO /SNO	Effect	Target	Mechanism	Refs.
NO	Anti-inflammatory	TNFα (kidney, liver)	Inhibiting protein expression	[9,10]
		IL-1 (liver)		[11]
		MIP-1/2 (myocardium)		[12]
		P-selectin (neutrophil)		[13]
	Antioxidant	ROS (ubiquitous)	Scavenging oxygen radical	[14,15,16,17,18]
	Regulation of cell signaling	p38 MAPK (hepatocyte)	Activating signal pathway	[19]
		NF-κB (neuron)	Inhibiting signal pathway	[20,21]
		AP-1 (neuron)		[22,23]
SNO	Protective	α1-PI (liver)	Inhibiting hepatocyte apoptosis	[24]
		CypD (heart)	Inhibiting mPTP opening	[25]
		PHB (neuron)	(not known)	[26,27]
	Injurious	ASK1 (hippocampus)	Inducing apoptosis	[28,29]
		GluR6 (hippocampus)	Increasing NO excessively	[30,31]
		PDI (astrocytes)	Forming protein aggregation	[32]
